# Study Protocol: Randomised Controlled Trial Assessing the Efficacy of Strategies Involving Self-Sampling in Cervical Cancer Screening

**DOI:** 10.3389/ijph.2022.1604284

**Published:** 2022-02-24

**Authors:** Caroline Lefeuvre, Hélène De Pauw, Anne-Sophie Le Duc Banaszuk, Adeline Pivert, Alexandra Ducancelle, Franck Rexand-Galais, Marc Arbyn

**Affiliations:** ^1^ Université d’Angers, HIFIH, UPRES EA 3859, Angers, France; ^2^ Département de Biologie des Agents Infectieux, Laboratoire de Virologie, Centre Hospitalier Universitaire d’Angers, Angers, France; ^3^ Unit of Cancer Epidemiology, Belgian Cancer Centre, Sciensano, Brussels, Belgium; ^4^ Centre Régional de Coordination de Dépistages des Cancers Pays de la Loire (CRCDC Pays de La Loire), Angers, France; ^5^ Laboratoire CLiPsy (BePsyLab), Faculté des Lettres, Langues et Sciences Humaines, Département Psychologie, Maison de la Recherche Germaine Tillon, Université d'Angers, Angers, France; ^6^ Department of Human Structure and Repair, Faculty of Medicine and Health Sciences, University Ghent, Ghent, Belgium

**Keywords:** cervical cancer, screening coverage, under-screened women, urinary self-sampling, vaginal self-sampling, cancer screening test, semi-structured interviews, randomised controlled trial

## Abstract

**Objectives:** The cervical cancer screening coverage remains moderate (60%) in France. The aim of the study is to evaluate the efficacy of two experimental invitation strategies (offer of urine or vaginal self-sampling kits) to reach under-screened populations and compare them with the current invitation strategy in rural departments (low medical density and low participation rate) in France.

**Methods:** The study is a randomised controlled trial with three arms: a control arm (conventional invitation letter) and two experimental arms (mailing of a urine or vaginal self-sampling kit). The target population includes women aged 30–65 years, who had no screening test recorded since more than 4 years and who did not respond to an invitation letter within 12 months before. The primary outcome measure is the participation rate in each arm. A team of psychologists will also investigate attitudes and experiences by semi-structured/focus-group interviews with voluntary CapU4 participants and with health professionals.

**Result and conclusion:** CapU4 will identify effective strategies to reach women not responding to current screening invitations and will generate information about acceptance of self-sampling among women and health professionals.

## Introduction

In 2018, cervical cancer (CC) ranked fourth in terms of cancer incidence and was the fourth most frequent cause of cancer-related mortality among women worldwide [1]. In France, it was the 12th most common cancer with 2,900 new cases per year and the 12th most common cancer for mortality (about 1,100 deaths per year) [2]. In Europe, CC screening over the last 50 years has significantly reduced the incidence and mortality associated with CC [3, 4]. This decrease is attributed to the widespread use of CC screening with a Pap smear [5]. At this time, despite the campaigns and letters of encouragement to have the cervical sample, the screening coverage remains moderate (around 60%) in France [2]. Various barriers to women’s participation have already been examined in previous studies: fear for the speculum examination, discomfort with the gynaecological examination, a previous negative experience, unequal access to gynaecological follow-up, time constraints, fear of the result and treatments and low perceived risk of CC [6–8].

The third French Cancer Plan (2014–2019) aimed to extend organised cervical cancer screening piloted in 12 departments including the Maine-et-Loire. The long-term objective was to reduce incidence and mortality by 30% in 10 years, by achieving 80% screening coverage and by making screening more accessible to vulnerable populations (economic precariousness because they benefit from supplementary universal health care coverage, medically desertified places with unequal access to gynaecological follow-up or the lack of a gynaecologist or family physician, etc.) [9]. In July 2019, the French National Authority for Health (Haute Autorité de Santé, HAS) proposed a new national strategy involving Human Papillomavirus (HPV)-based screening for women aged 30–65 years at an interval of 5 years, with the option of vaginal self-sampling as part of a reminder. For women aged 25 to 29, screening is still based on cytological examination of a Pap smear every 3 years after two consecutive annual smears (previous recommendation for all women aged 25–65 years) [10].

Since 2020, the organised screening has been extended nationally. It includes sending invitations to have a cervical specimen taken by a health professional sent to women who have not been screened for more than 3 years, with reminders sent again if no response registered 12 months after the first invitation, and a follow-up of all women for whose screening test result is abnormal (pathological smear or positive HPV test) [11, 12].

Additional interventions are being considered to increase screening coverage, such as offering self-sampling kits to non-responders. Systematic reviews have shown that HPV DNA testing using an appropriate PCR-based assay on a self-taken vaginal sample is as accurate in detecting cervical precancer as HPV testing on a clinician-taken sample [13]. Moreover, meta-analyses of randomised participation trials have demonstrated that sending self-sampling kits is more effective in reaching under-screened populations than conventional invitations to have a screening specimen taken by a clinician. However, the absolute participation rates are highly variable among studies [14]. HPV testing can also be performed on first-void urine [15], which is less invasive and might be even more acceptable than vaginal self-sampling for women reluctant to undergo gynaecologic examinations. The clinical accuracy of HPV testing on urine and on vaginal samples was investigated in the VALHUDES trial (VALidation of HUman papillomavirus assays and collection DEvices for Self-samples and urine samples) [16]. However, no data exist today indicating that sending urine collection kits may be more effective than sending vaginal self-collection kits or conventional invitations. HPV testing on urine is currently only proposed in research protocols [17–19]. Between 2016 and 2018, the CapU3 study sent approximately 13,000 urine collection kits to women living in the Department of Maine-et-Loire, aged 35–65 years, who did not have screening record since 7 years ago or longer [20]. In the routine screening programme, low response rates were observed among women who often remain indifferent to re-invitations sent by simple mail. In the CapU3 study, we noted higher responses (participation rate of 15.4%) when urine collection kits were sent to non-participants in medically underserved areas [20]. However, the CapU3 trial did not include a comparison arm impeding to demonstrate evidence of comparative efficacy.

This article describes the protocol for a randomised trial in which we will assess the efficacy of two experimental invitation strategies (including self-sampling) to reach under-screened populations and compare them with the current invitation strategy in three rural departments (low medical density and low rate of smear participation) in France.

## Methods

### Objectives

The main objective of CapU4 study is to evaluate the effectiveness of two experimental invitation strategies (urine or vaginal self-sampling) to reach under-screened populations and compare them to the current invitation strategy in rural departments in France, and to improve the response rate among women aged 30–65 years (not screened over a period longer than the recommended screening interval) who did not respond to a conventional prior invitation. The trial will assess the response to alternative invitation strategies and answer the question of whether experimental interventions including sending of self-sampling devices are more effective in generating greater participation in screening than sending conventional reminder letters. The trial will be conducted in different geographical areas to verify whether findings are reproducible in diverse regions.

The secondary objectives are to collect the barriers and levers that a self-sampling received at home can provide; to collect the barriers and levers to CC screening from health professionals [general practitioners (GPs), midwives and gynaecologists]; to assess compliance with gynaecological follow-up among women with a positive HPV test and to map the distribution of genotypes.

### Trial Design

**FIGURE 1:**
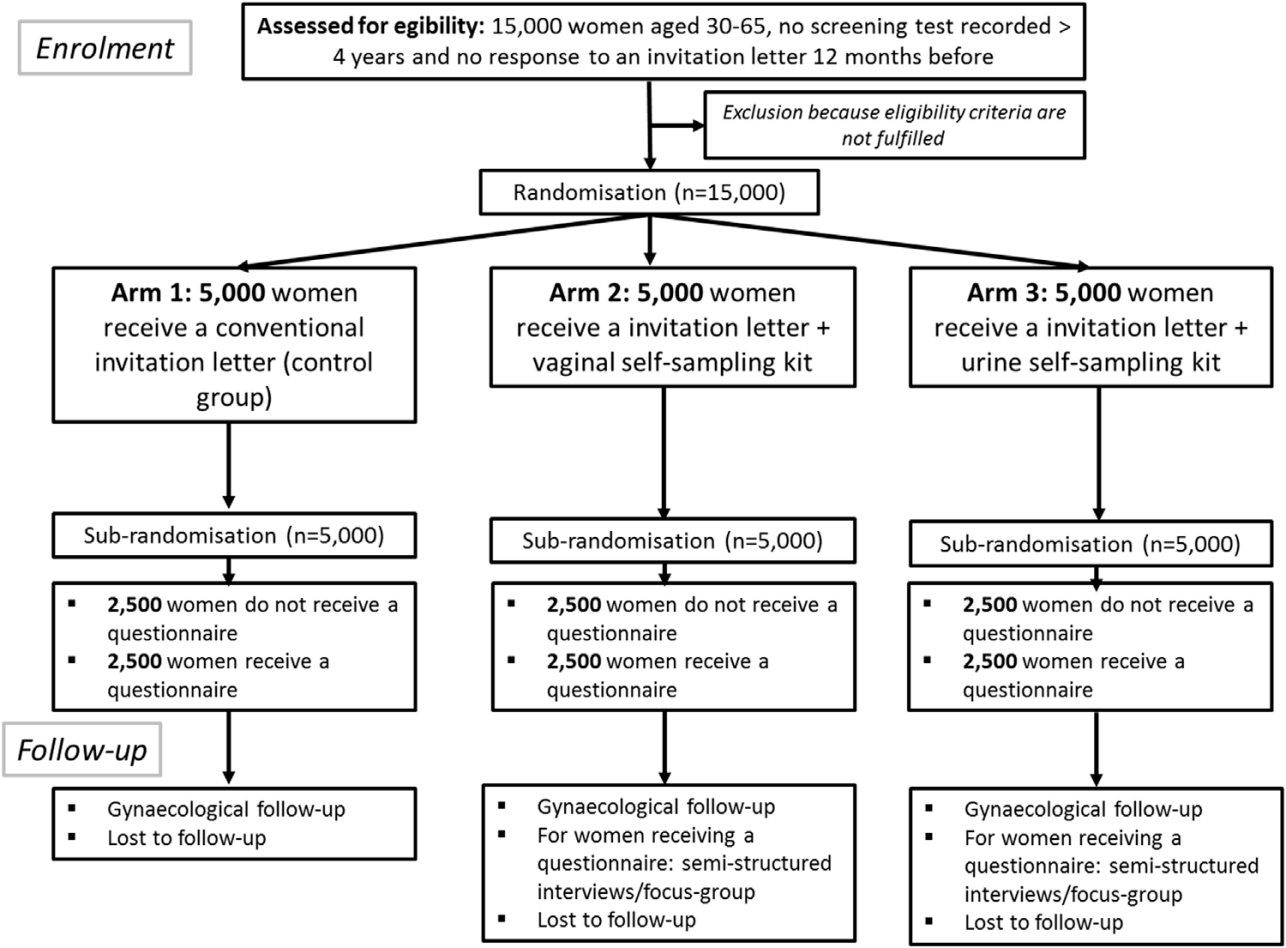
Flow chart describing the study. The CapU4 protocol–France—2022–2024.

**FIGURE 2:**
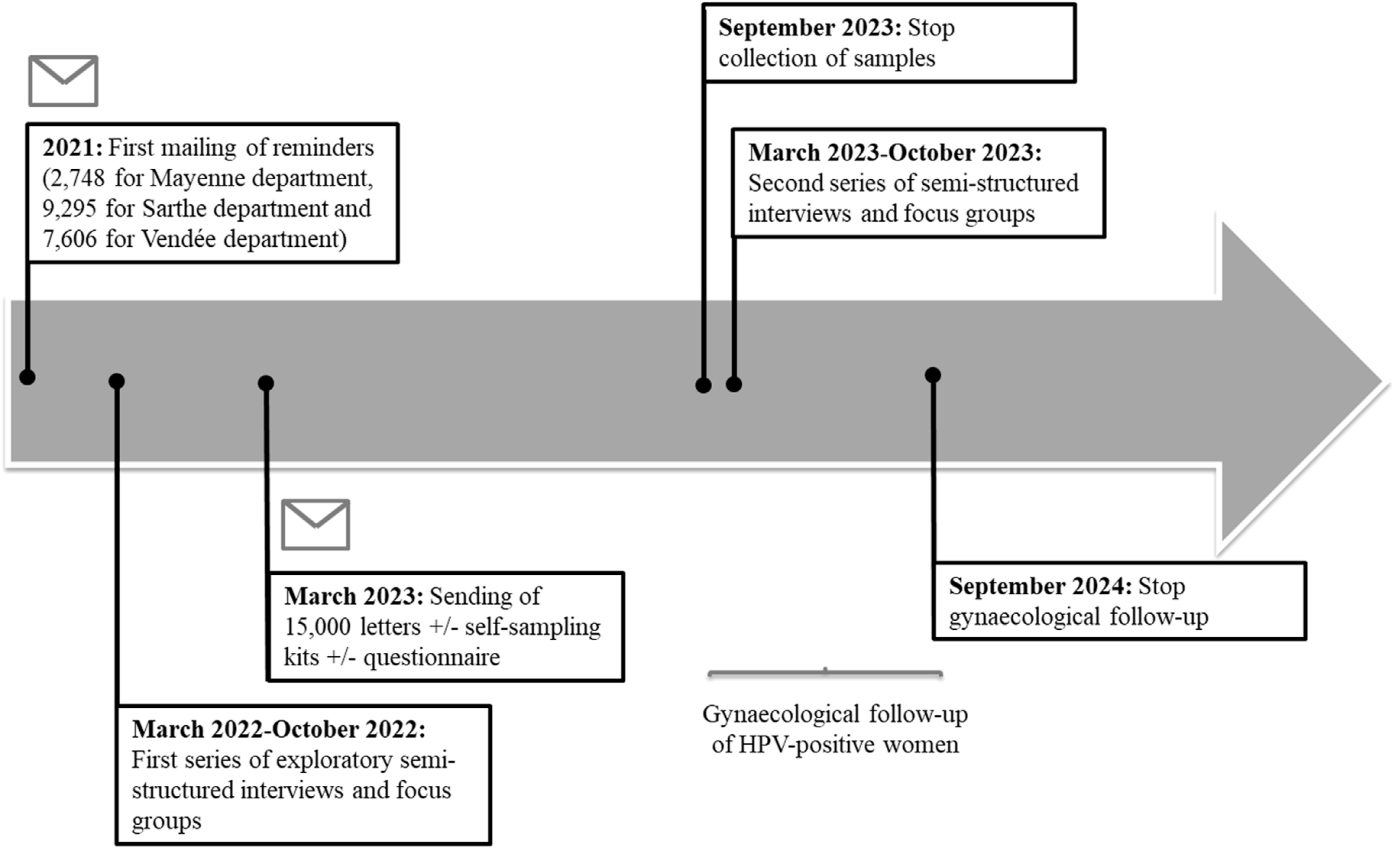
Timeline of study recruitment, HPV testing, gynaecological follow-up and semi-structured interviews/focus groups. The regional deployment of the national organized cervical cancer screening program began in Pays de la Loire in 2020. The sending of the 15,000 conventional letters associated or not with a self-sampling kit or a questionnaire will take place in March 2023. A first series of semi-structured interviews and exploratory focus groups was carried out in 2022. A second series took place in 2023. Samples will be collected until September 2023. Gynaecological follow-up of women (collection of cytological results of smears and histological results following a possible colposcopy) will continue until September 2024. The CapU4 protocol—France—2022–2024.

The CapU4 study is a randomised controlled trial (1:1:1) with three arms with two experimental interventions and one control arm. Figure 1 illustrates the trial design and the SPIRIT checklist is detailed in Supplementary Material. Figure 2 summarises the timeline of the study.

The three arms will be: 1) women receive a conventional invitation letter sent by post to the home address of eligible women recommending them to make an appointment to a doctor or a midwife for the collection of a cervical specimen; 2) eligible women receive at their home address an invitation letter with, in addition, a vaginal self-sampling kit; and 3) eligible women receive at their home address an invitation letter with, in addition, a urine collection kit. Half of the women will receive a questionnaire adapted according to the type of self-sampling or the control arm (Supplementary Material) after a sub-randomisation performed in each arm (Figure 1). A team of psychologists will investigate attitudes and experiences by semi-structured/focus-group interviews with voluntary CapU4 participants and with health professionals.

### Participants

The target population will be women aged between 30 and 65 years, living in the Departments of Mayenne, Sarthe and Vendée (Pays de la Loire, France) and who have not carried out a screening test (cytology of smear or HPV test) following a letter sent 12 months previously in 2021. Therefore, these are women whose last cervical sample was performed more than 4 years ago and who are reluctant to receive at least a first letter.

Non-inclusion criteria are: recent cervical sampling (less than 3 years old), women younger than 30 or older than 65 years, women who have had a hysterectomy, women with ongoing follow-up for a cervical lesion, women who are not members or beneficiaries of a social security system.

The target population will be defined by a query in the Centre Régional de Coordination de Dépistages des Cancers (CRCDC)—Pays de la Loire’s business software. CRCDC, which is a public health body governed by the French law on non-profit associations, provides colorectal, breast, and cervical cancer screenings in the Pays de la Loire region.

### Study Setting

The departments of Mayenne, Sarthe and Vendée were chosen because their participation rate for cervical smear screening is lower than the French national and the Pays de la Loire region average. These are rural territories known as medical desertification where access to care is limited due to low population density and larger distances to health services. The population of women aged between 30 and 65 years in Mayenne is 67,946, in Sarthe is 126,765, in Vendée is 157,050 and in Maine-et-Loire is 181,306 according to the French Public Health estimate for 2019. We will also be able to compare these three departments, in which organised screening is starting, with the Maine-et-Loire department which has been experimenting with this screening for 10 years.

### Interventions

The control arm corresponds to women receive a conventional invitation letter sent by post to the home address of eligible women recommending them to make an appointment to a doctor or a midwife for the collection of a cervical specimen. The two experimental interventions are: 1) eligible women receive at their home address a vaginal self-sampling kit (FLOQSwabs^®^ Copan Diagnostics, Brescia, Italy) in addition to the conventional invitation letter; and 2) eligible women receive at their home address a urine collection kit (Colli-Pee device, Novosanis, Wijnegem, Belgium) in addition to the conventional invitation letter.

With the conventional invitation letter, the women will receive an information brochure on the study (arms 2 and 3), a questionnaire (half of the population of the three arms), a self-sampling device with instruction (arms 2 and 3) and a prepaid return envelope. The women who will receive the self-sampling kit will place their prepaid return envelope in a post box.

### Outcomes

#### Primary Outcomes

Participation rates (number of responding/number of invited) in each arm, difference and ratios of participation rates between the control and the two intervention arms.

#### Secondary Outcomes

⁃ Contrast in participation between the 2 experimental arms and between women that received a questionnaire or not.
⁃ Screen test positivity rates (presence of high-risk HPV and distribution by HPV type and/or of abnormal cytology) in the respective arms and contrasts between arms.
⁃ Adherence to follow-up of screen-positive women and contrasts between arms.
⁃ Impact of covariates on the participation and adherence to follow-up (age, reimbursement status, geographical area).
⁃ The obstacles and levers emerging from the speech of the women and health professionals participating in the research will be analysed on the basis of a thematic analysis [21] integrating a specific focus on the indicators identified in previous studies (e.g., lack of time, discomfort with regard to the location or bad experience encountered during another type of examination, etc.). This study will be doubled by a categorical analysis based on Linguistic Inquiry and Word count which allows, among other things, to automatically categorise positive or negative emotions related to themes for example associated with the disease or beliefs [22].

### Sample Size

To demonstrate differences in response rates between the control arm (assumed to vary in the range 12–14%, based on average estimates in a French trial [23] and observations from a meta-analysis [14]) and response rates in the experimental arms (assumed to be 3%–7% higher), a sample size between 588 and 3,123 women per arm would be needed in 1:1:1 randomised trial, accepting a confidence level of 95% and a power of 90%. To estimate the compliance with further follow-up among women with a positive screening test with a 95% confidence interval (CI) width of 10%, assuming compliance rates ranging from 41% to 92% respectively lowest and highest rates observed in a meta-analysis [14], a sample size varying from 1,327 to 4,360 women is needed. By pooling the two experimental arms, it will be possible to obtain more precise (more narrow CIs) estimates of the adherence to further follow-up among women with an HPV-positive result on a self-sample. To conclude enrolment of 3 × 5,000 women will allow sufficient power to demonstrate higher efficacy of the experimental compared to the control arm and to reach sufficient precision to estimate follow-up compliance.

### Randomisation

Randomisation will be performed using the Stata 16.0 random number generator (College Station, TX, United States).

### Data Management

The target population will extract thanks to a query in the CRCDC-Pays de la Loire’s business software called Zeus d’Osi-Santé. The list of patients will be included first and last names, dates of birth, addresses, and social security numbers to avoid confusion and duplications. Results of HPV self-sampling test and questionnaire data will be compiled in a restricted access file. The CRCDC will ensure the completeness of data collection for women who have visited their physician/midwife for gynaecological sampling through the collection of reimbursement data and screening test results from cytopathology or medical laboratories.

The interviews and focus groups are digitally recorded after information about the purposes of the research and the completion of a consent form.

### Statistical Methods

The Unit of Cancer Epidemiology (Sciensano, Brussels, Belgium) will perform the statistical analyses of a pseudo-anonymised data file containing records of participating women.

Contrasts in response in women included in the self-sampling arms versus the control arm will be evaluated as relative participation (proportion participating in one arm/proportion participating in another arm) and participation differences (proportion participating in one arm - proportion participating in another arm). A positive response is defined as a woman who has received a screening test: a cervical smear (in the control arm) and a vaginal or urine self-sampling (experimental arm) within 6 months after the offered intervention. The cumulative response rate over time (total number of responses observed by time of observation) will be assessed graphically using Kaplan-Meier curves. Reaching a plateau will indicate that the “invitation effect” is ending and this information will help to define the observation period required to observe the effect of an intervention in the future. Inter-arm contrasts in cumulative participation will be evaluated by Cox’ proportional hazard regression.

Two types of analysis will be performed: per protocol analysis where only responses foreseen in the respective arm will be considered, whereas in the intention to treat analysis also women that are screened not according to the foreseen intervention will be considered as well (also those performed on samples taken by a clinician in the self-sampling arms). The influence of age category and geographical area will be assessed using by Pearson’s *χ* ^2^ statistic and logistic regression.

In addition, knowledge, attitudes, opinions and experiences of participating women, as well as the variation by age, geographical area (including the deprivation index corresponding with the statistical sector where the woman is domiciled) and previous screening history will be assessed in the returned questionnaires.

All statistical analyses will be performed using Stata version 16 (StataCorp LLC, College Station TX, United States). The *p*-value for statistical significance will be defined at ≤0.05.

### Additional Analyses

#### Laboratory Testing and Gynaecological Follow-Up

The vaginal and urinary self-samples will be analysed at the virology laboratory of the Angers University Hospital using real-time PCR for the search of oncogenic HPV with extended genotyping (BD Onclarity™ HPV test, BD Diagnostics, Burlington, NC). The BD Onclarity™ HPV test provides results for six individual high-risk HPV genotypes (16, 18, 31, 45, 51 and 52), with the remaining eight high-risk genotypes reported in three small groups: (33, 58), (35, 39, 68) and (56, 59, 66). The Onclarity™ assay fulfills requirements for HPV tests in the context of cervical cancer screening [24, 25]. Negative results will be sent to the woman, her GP and the CRCDC. Positive results with oncogenic HPV will be sent to the GP and the CRCDC. The woman will receive a letter urging her to go to her GP to get her result. The CRCDC will follow up with the doctor and the woman if she had not had a smear after 6 months. Reminders will be regularly until the follow-up examination is carried out.

#### Attitudes and Acceptance by Women

A questionnaire will be sent to half of the population of the three arms to assess the reasons for not performing a cervical smear. The benefits of performing self-sampling will concern arms 2 and 3 with the self-samplings kits (see Supplementary Material).

Then, in order to understand the obstacles of the target population regarding screening, semi-structured interviews will be first conducted by psychologists [26, 27]. The interviews will be recruited among the women who performed the self-sampling and who have agreed to leave their contact information. A random draw based on socio-demographic and territorial characteristics will then be carried out to avoid the over-representation of certain populations. Special attention will be paid to the population characterised as under-screened (over 50 years old) and to vulnerable populations (economic precariousness because they benefit from complementary health care, medically desertified places, etc.). These interviews may be conducted face-to-face or remotely depending on the woman’s medical and geographical situation. The objective will be to determine the motivations of the woman who returned a self-sample, stopping on the cognitive path from the knowledge of the device to the pre-diagnosis and its consequences but also what they do with the result received. Indeed, the reception of a negative, unsatisfactory or positive result may not lead to a medical consultation, even though it would be recommended. The semi-directive interview will also allow gathering the experience of the sample collection process to measure satisfaction and assess the value given to the information received.

After the first series of exploratory semi-structured interviews (envisaged number of interviews: 6), focus groups discussions will be implemented. According to Kitzinger and al. [28, 29], focus groups will bring together between five to eight participants and, according to Hennink et al. [30], their number will be between 6 and 8. The use of focus groups is particularly useful in understanding similarities and differences in the thoughts, views and emotions of participants [31]. This method is therefore indicated for the purpose of the study and allows the obtaining of additional information for semi-structured interviews. This qualitative technique has been chosen in numerous recent studies relating to self-sampling and cervical cancer [32–34]. Its use will give the possibility of international comparisons. Third, these focus groups will be followed by a new series of semi-structured interviews to investigate elements that emerge from the focus groups and require individual exploration. Raising the number of interviews to 10–14, this series will ensure that data saturation is achieved.

#### Attitude of Health Professionals

In order to identify the obstacles and levers of primary care health professionals (GPs, midwives and gynaecologists), psychologists will organise semi-structured interviews, depending on the professional and territorial constraints of the surveys, in particular to take into account the importance of recruiting professionals practicing in rural or semi-rural areas.

The health professionals will be informed in advance by mail of the conditions of the research and will be offered to participate in an interview. They will be informed that they will be contacted by telephone after the letter has been sent in order to allow for a further exchange of information and to agree, if they wish, on the conditions for the implementation of this interview. The aim of these semi-structured interviews will be to understand the different situations encountered, the resources mobilized, the difficulties overcome and those remaining to improve both the system and the modalities (sending the screening kit at home or to the doctor’s office), based on collective exchanges. The interviews will be conducted in the number necessary to reach saturation of responses [35].

Semi-structured interviews and focus groups will be recorded and transcribed. They will be analysed using conventional qualitative content analysis [36].

### Ethical Consideration

The CapU4 study protocol received approval from the French “Sud-Est I Ethics Committee” (2021-123, November 25, 2021, France). The supply and use of these records for the programme have been submitted to the Commission Nationale de l’Informatique et des Libertés, French National Data Protection Authority in September 2021 (ref. 2223607v0). Women’s return of the vaginal and urinary self-samples will constitute consent for HPV testing. Written informed consent to the audio recording of the interviews and anonymised digital transcripts will be obtained from the women who will participate in the individual semi-structured interviews and/or focus groups.

## Discussion

CapU4 will for the first time compare directly two experimental invitational strategies (including self-sampling of vaginal specimens and collection of first-void urine) with the current procedure including traditional reminder letters with the aim to increase participation in cervical cancer screening. The trial will take place in three rural medically under-served departments, in France, with low screening coverage.

Based on the results of the CapU3 study, we expect that the participation in the self-sampling arms will be between 15% and 20% [20] which should be higher than the response to a routine reminder letter inviting to contact a health professional for the collection of a cervical smear. A recent meta-analysis has shown that strategies including sending self-sampling kits to the women’s home address are more effective than “opt-in” strategies where women have to request a self-sampling kit or routine letters inviting women to have a cervical specimen by a clinician [14]. Another French study also demonstrates that offering a return-mail kit for in-home vaginal self-sampling is more effective and cost-effective than a recall letter in increasing participation in cervical cancer screening [23].

Self-sampling is becoming increasingly important and applicable to supplement organised screening programme. Vaginal self-sampling modalities are recognised in France and recommended for non-participating women since 2019 [10].

Several studies have been set up recently that compares the clinical accuracy of HPV testing on diverse types of self-samples (vaginal, first-void urine) with HPV testing on cervical samples taken by a clinician [16, 37–39].

The areas of the three departments of Sarthe, Mayenne and Vendée are for the most part in the area of medical priority intervention. The healthcare context linked to the COVID-19 pandemic, with three periods of confinement, exacerbates inequalities in access to care with an increase in territorial inequalities due to transport restrictions. The option to collect a self-sample at home may address this accessibility problem. Home-based sample collection should be done in accordance with national recommendations and as part of an organised screening programme that ensures the follow-up of screen-positive women. We will be able to assess accessibility to health services by correlating participation with the density of health services corresponding with the statistical sector.

CapU4 will also monitor the follow-up of screen-positive women in each of the arms through the regional screening centre (CRCDC), with particular attention to the adherence of to the recommendation to have a cervical smear to triage women with an HPV-positive self-sample. The CapU3 study had enabled more than 92% of the women screened to have gynaecological follow-up [20]. The same result is expected in this study.

Strategies that overcome barriers to women’s participation are imperative to improve coverage of cervical screening programmes, but these new strategies also need to be evaluated. The knowledge of obstacles to CC screening will make it possible to optimise the modalities (instructions for use, receipt of results mail) measuring women’s satisfaction. Templates from other countries or regions and suggestions captured from focus-group discussions could help to adapt communication strategies and improve the participation rate of the target population.

This analysis will make it possible to refine the instructions for use and access methods for self-sampling for deployment to all follow-up campaigns in the region and even nationally.

The CapU4 will be completed with an economic analysis where we will assess the incremental cost-effectiveness (extra cost per extra screened woman in the two self-sampling arms versus control arm and in the urinary versus vaginal arms).

Finally, from the qualitative information collected in the in-depth interviews, we will be able to identify the effective strategies for reminders (by simple mail, urinary or vaginal self-sampling kits) for both professionals and the general public.
