# Corrigendum: Study Protocol: Randomised Controlled Trial Assessing the Efficacy of Strategies Involving Self-Sampling in Cervical Cancer Screening

**DOI:** 10.3389/ijph.2024.1607299

**Published:** 2024-04-18

**Authors:** Caroline Lefeuvre, Hélène De Pauw, Anne-Sophie Le Duc Banaszuk, Adeline Pivert, Alexandra Ducancelle, Franck Rexand-Galais, Marc Arbyn

**Affiliations:** ^1^ Université d’Angers, HIFIH, UPRES EA 3859, Angers, France; ^2^ Département de Biologie des Agents Infectieux, Laboratoire de Virologie, Centre Hospitalier Universitaire d’Angers, Angers, France; ^3^ Unit of Cancer Epidemiology, Belgian Cancer Centre, Sciensano, Brussels, Belgium; ^4^ Centre Régional de Coordination de Dépistages des Cancers Pays de la Loire (CRCDC Pays de La Loire), Angers, France; ^5^ Laboratoire CLiPsy (BePsyLab), Faculté des Lettres, Langues et Sciences Humaines, Département Psychologie, Maison de la Recherche Germaine Tillon, Université d’Angers, Angers, France; ^6^ Department of Human Structure and Repair, Faculty of Medicine and Health Sciences, University Ghent, Ghent, Belgium

**Keywords:** cervical cancer, screening coverage, under-screened women, urinary self-sampling, vaginal self-sampling, cancer screening test, semi-structured interviews, randomised controlled trial

There was a mistake in the caption for **Figure 1** as published. The addition of the Vendée department delayed the start of the study and changed the timeline of study. This caption previously stated:

“Flow chart describing the study. The CapU4 protocol–France—2022–2023.”

The corrected caption appears below:

“Flow chart describing the study. The CapU4 protocol–France—2022–2024.”

There was a mistake in the caption for Figure 2 as published. The addition of the Vendée department delayed the start of the study and changed the timeline of study. The 15,000 letters were sent in March 2023 (not January 2022). A second series of semi-structured interviews and focus groups took place in 2023, in addition to 2022. This caption previously stated:

**FIGURE 2:**
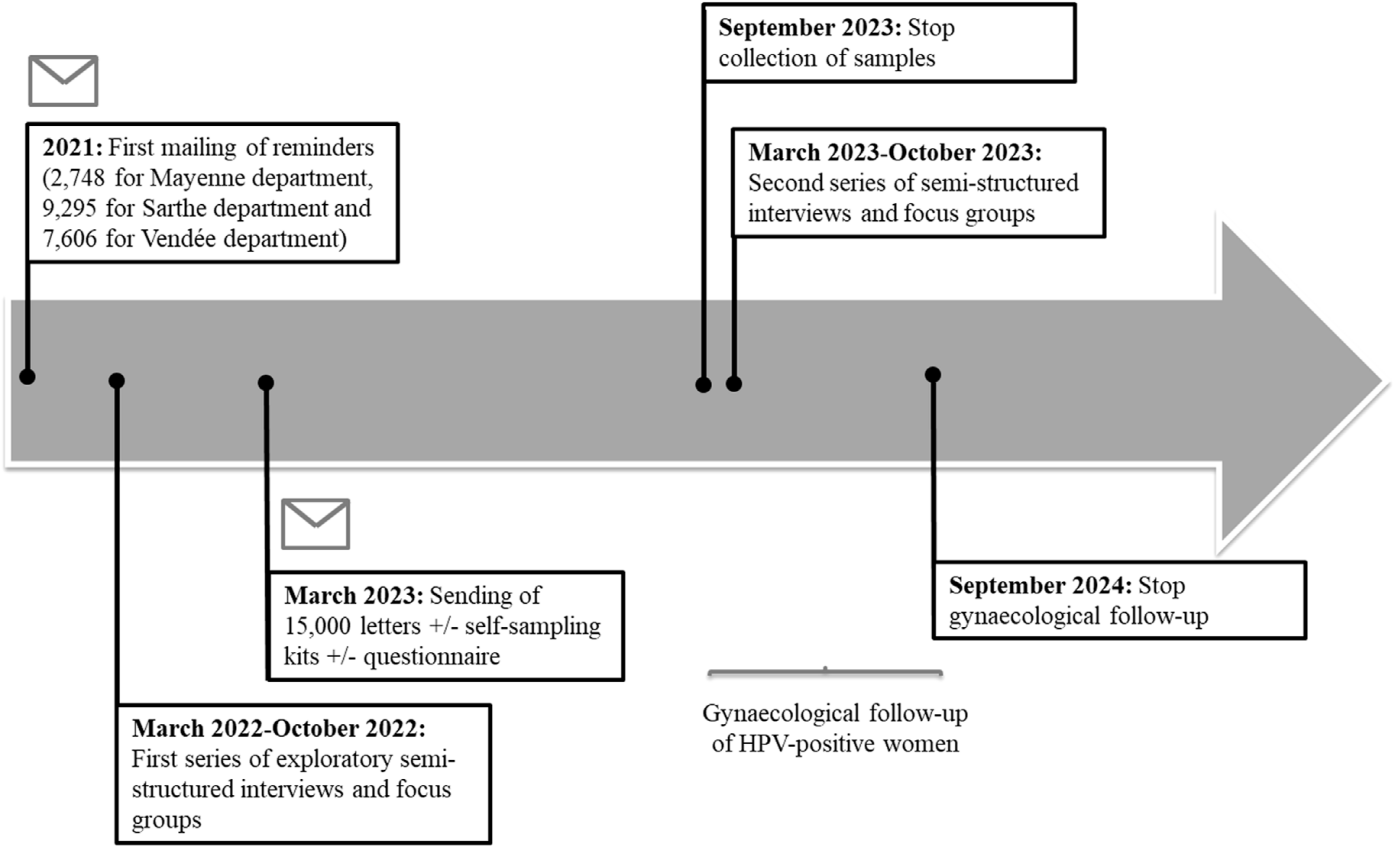
Timeline of study recruitment, HPV testing, gynaecological follow-up and semi-structured interviews/focus groups. The regional deployment of the national organized cervical cancer screening program began in Pays de la Loire in 2020. The sending of the 15,000 conventional letters associated or not with a self-sampling kit or a questionnaire will take place in March 2023. A first series of semi-structured interviews and exploratory focus groups was carried out in 2022. A second series took place in 2023. Samples will be collected until September 2023. Gynaecological follow-up of women (collection of cytological results of smears and histological results following a possible colposcopy) will continue until September 2024. The CapU4 protocol—France—2022–2024.

“Timeline of study recruitment, HPV testing, gynaecological follow-up and semi-structured interviews/focus groups. The regional deployment of the national organized cervical cancer screening program began in Pays de la Loire in 2020. Extractions will be started in September 2021 until saturation of the number of women planned in the study, i.e. 15,000 women randomly distributed in three arms of equivalent number. The sending of the 15,000 conventional letters associated or not with a self-sampling kit or a questionnaire will take place in January 2022. Semi-structured interviews and focus groups will only be carried out in 2022 after the collection of samples, scheduled for July 2022, has been completed. Gynaecological follow-up of women (collection of cytological results of smears and histological results following a possible colposcopy) will continue until June 2023. The CapU4 protocol—France—2022–2023.”

The corrected caption appears below:

“Timeline of study recruitment, HPV testing, gynaecological follow-up and semi-structured interviews/focus groups. The regional deployment of the national organized cervical cancer screening program began in Pays de la Loire in 2020. The sending of the 15,000 conventional letters associated or not with a self-sampling kit or a questionnaire will take place in March 2023. A first series of semi-structured interviews and exploratory focus groups was carried out in 2022. A second series took place in 2023. Samples will be collected until September 2023. Gynaecological follow-up of women (collection of cytological results of smears and histological results following a possible colposcopy) will continue until September 2024. The CapU4 protocol—France—2022–2024.”

There was a mistake in Figure 2 as published. The addition of the Vendée department delayed the start of the study and changed the timeline of study. The department of Vendée was added to the Mayenne and Sarthe departments in order to reach the planned 15,000 invitations. The 15,000 letters were sent in March 2023 (not January 2022). A second series of semi-structured interviews and focus groups took place in 2023, in addition to 2022. The corrected Figure 2 appears below.

In the first published version of the article there was an error. The department of Vendée (Pays de la Loire region, France) was not mentioned in the text. The department of Vendée was added to the Mayenne and Sarthe departments in order to reach the planned 15,000 invitations.

A correction has been made to **Introduction**, *paragraph 5*. This sentence previously stated:

“This article describes the protocol for a randomised trial in which we will assess the efficacy of two experimental invitation strategies (including self-sampling) to reach under-screened populations and compare them with the current invitation strategy in two rural departments (low medical density and low rate of smear participation) in France.”

The corrected sentence appears below:

“This article describes the protocol for a randomised trial in which we will assess the efficacy of two experimental invitation strategies (including self-sampling) to reach under-screened populations and compare them with the current invitation strategy in three rural departments (low medical density and low rate of smear participation) in France.”

A correction has been made to **Methods**, Participants, *paragraph 1*. This sentence previously stated:

“The target population will be women aged between 30 and 65 years, living in the Departments of Mayenne and Sarthe (Pays de la Loire, France) and who have not carried out a screening test (cytology of smear or HPV test) following a letter sent 12 months previously in 2020.”

The corrected sentence appears below:

“The target population will be women aged between 30 and 65 years, living in the Departments of Mayenne, Sarthe and Vendée (Pays de la Loire, France) and who have not carried out a screening test (cytology of smear or HPV test) following a letter sent 12 months previously in 2021.”

A correction has been made to **Methods**, Study setting. This sentences previously stated:

“The departments of Mayenne and Sarthe were chosen because their participation rate for cervical smear screening is lower than the French national and the Pays de la Loire region average. These are rural territories known as medical desertification where access to care is limited due to low population density and larger distances to health services. The population of women aged between 30 and 65 years in Mayenne is 67,946, in Sarthe is 126,765 and in Maine-et-Loire is 181,306 according to the French Public Health estimate for 2019. We will also be able to compare these two departments, in which organised screening is starting, with the Maine-et-Loire department which has been experimenting with this screening for 10 years.”

The corrected sentences appear below:

“The departments of Mayenne, Sarthe and Vendée were chosen because their participation rate for cervical smear screening is lower than the French national and the Pays de la Loire region average. These are rural territories known as medical desertification where access to care is limited due to low population density and larger distances to health services. The population of women aged between 30 and 65 years in Mayenne is 67,946, in Sarthe is 126,765, in Vendée is 157,050 and in Maine-et-Loire is 181,306 according to the French Public Health estimate for 2019. We will also be able to compare these three departments, in which organised screening is starting, with the Maine-et- Loire department which has been experimenting with this screening for 10 years.”

A correction has been made to **Discussion**, *paragraph 1*. This sentence previously stated:

“The trial will take place in two rural medically under-served departments, in France, with low screening coverage.”

The corrected sentence appears below:

“The trial will take place in three rural medically under-served departments, in France, with low screening coverage.”

A correction has been made to **Discussion**, *paragraph 5*. This sentence previously stated:

“The areas of the two departments of Sarthe and Mayenne are for the most part in the area of medical priority intervention.”

The corrected sentence appears below:

“The areas of the three departments of Sarthe, Mayenne and Vendée are for the most part in the area of medical priority intervention.”

The authors apologize for these errors and state that this does not change the scientific conclusions of the article in any way. The first published incorrect version of the article has been updated.

