# Supplementary material for: Study Protocol: Randomised Controlled Trial Assessing the Efficacy of Strategies Involving Self-Sampling in Cervical Cancer Screening
Source: Int J Public Health. 2022 Feb 24;67:1604284. doi: 10.3389/ijph.2022.1604284 (PMC8907121; doi:10.3389/ijph.2022.1604284)
Supplement: Supplementary file 1 [file DataSheet2.doc]

**Supplementary Material**


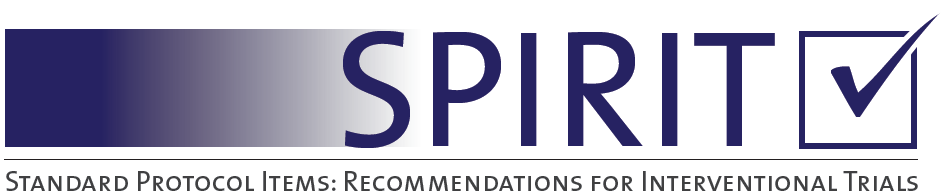


SPIRIT 2013 Checklist: Recommended items to address in a clinical trial protocol and related documents*

| Section/item | ItemNo | Description |  |
| --- | --- | --- | --- |
| **Administrative information** | | |  |
| Title | 1 | Descriptive title identifying the study design, population, interventions, and, if applicable, trial acronym | CapU4, a randomised controlled trial assessing the efficacy of strategies involving self-sampling in cervical cancer screening |
| Trial registration | 2a | Trial identifier and registry name. If not yet registered, name of intended registry | CapU4 («CapSanté» Urinary study number Four);  Registration trial in progress |
|  | 2b | All items from the World Health Organization Trial Registration Data Set | IDRCB number: 2021-A01660-41.  Trial registration on ANSM (Agence Nationale du Médicament des Produits de Santé) web site (number 6892111). |
| Protocol version | 3 | Date and version identifier | 05/07/2021, version 1 |
| Funding | 4 | Sources and types of financial, material, and other support | The CapU4 study is funded by the French National Cancer Institute (INCa) (attribution number 2021-158). |
| Roles and responsibilities | 5a | Names, affiliations, and roles of protocol contributors | - Dr. Anne-Sophie Banaszuk, Centre Régional de Coordination de Dépistages des Cancers (CRCDC) - Pays de la Loire, France : trial sponsor - Dr. Caroline Lefeuvre and Pr. Alexandra Ducancelle, Laboratory of virology, Angers University Hospital, France: analysis of vaginal and urinary self-samples - Dr. Franck Rexand Galais, BePsyLab laboratory, University of Angers, France: psychologists conducting semi-structured interviews and focus-groups - Dr. Marc Arbyn and Dr. Hélène De Pauw, Unit of Cancer Epidemiology (Sciensano, Brussels, Belgium): statistical analysis |
|  | 5b | Name and contact information for the trial sponsor | Centre Régional de Coordination de Dépistages des Cancers (CRCDC) - Pays de la Loire, France  Dr Anne-Sophie Banaszuk  5 rue des Basses Fouassières  49000 Angers |
|  | 5c | Role of study sponsor and funders, if any, in study design; collection, management, analysis, and interpretation of data; writing of the report; and the decision to submit the report for publication, including whether they will have ultimate authority over any of these activities | The funder was not involved in the study design, the writing of this article or the decision to submit it for publication. |
|  | 5d | Composition, roles, and responsibilities of the coordinating centre, steering committee, endpoint adjudication committee, data management team, and other individuals or groups overseeing the trial, if applicable (see Item 21a for data monitoring committee) | The CRCDC Pays de la Loire (screening organisation), under the coordination of Dr. Anne-Sophie Banaszuk, hereinafter referred to as the "Coordinating Investigator", is the sponsor of the CapU4 Study. The CRCDC will be in charge of the necessary queries in its software, the sending of the different invitations for each arm and the follow-up of the women taking part in the research. It will collect the answers to the questionnaire. |
| Introduction |  |  |  |
| Background and rationale | 6a | Description of research question and justification for undertaking the trial, including summary of relevant studies (published and unpublished) examining benefits and harms for each intervention | Research question: does the offer of self-sampling kits result in higher attendance to cervical cancer screening compared to sending invitation letters which recommend women to contact a health professional for taking a cervical specimen?  Systematic reviews indicate that HPV testing on vaginal specimens taken by the woman her-self is as accurate to detect cervical precancer as HPV testing of cervical specimens collected by a clinician, under the condition that a validated PCR-based HPV assay is used. Similar results are shown from recent studies where HPV testing was performed on first-void urine collected with an appropriate device and transport medium. A recent meta-analysis of randomised trials showed higher response rates when under-screened women receive a self-sampling kit at home compared to traditional invitation or reminder letters. However, the absolute participation rates are highly variable among studies. Whereas qualitative research indicates that women prefer collection of urine rather than a vaginal self-sample, no data are available that the offer of urine kits would result in higher participation among women who do not participate regularly in cervical cancer screening. |
|  | 6b | Explanation for choice of comparators | Two experimental arms: 1) women receive a vaginal self-collection kit, 2) women receive a urine collection device and one control arm: women receive the routine used invitation letter recommending collection of a cervical specimen by a physician of choice. |
| Objectives | 7 | Specific objectives or hypotheses | - To evaluate the effectiveness of two experimental invitation strategies (urine or vaginal self-sampling) to reach under-screened populations and compare them to the current invitation strategy in rural departments in France. - To improve the response rate among women aged 30 to 65 years (not screened over a period longer than the recommended screening interval) who did not respond to a conventional prior invitation. |
| Trial design | 8 | Description of trial design including type of trial (eg, parallel group, crossover, factorial, single group), allocation ratio, and framework (eg, superiority, equivalence, noninferiority, exploratory) | 1:1:1 randomised population-based participation trial, with 2 experimental and 1 control arm, with in each arm a 1:1 sub-randomisation with subgroup A receiving a questionnaire and subgroup B receiving no questionnaire. |
| Methods: Participants, interventions, and outcomes | | |  |
| Study setting | 9 | Description of study settings (eg, community clinic, academic hospital) and list of countries where data will be collected. Reference to where list of study sites can be obtained | The study takes place in the Departments of Mayenne and Sarthe (Pays de la Loire, France) because their participation rate for cervical smear screening is lower than the French national and the Pays de la Loire region average. |
| Eligibility criteria | 10 | Inclusion and exclusion criteria for participants. If applicable, eligibility criteria for study centres and individuals who will perform the interventions (eg, surgeons, psychotherapists) | Inclusion criteria: women aged between 30 and 65 years, living in the Departments of Mayenne and Sarthe (Pays de la Loire, France) and who have not carried out a screening test (cytology of smear or HPV test) following a letter sent 12 months previously in 2020.  Non-inclusion criteria: recent cervical sampling (less than three years old), women younger than 30 or older than 65 years, women who have had a hysterectomy, women with ongoing follow-up for a cervical lesion, women who are not members or beneficiaries of a social security system. |
| Interventions | 11a | Interventions for each group with sufficient detail to allow replication, including how and when they will be administered | The two experimental interventions are: 1) eligible women receive at their home address a vaginal self-sampling kit in addition to the conventional invitation letter; and 2) eligible women receive at their home address a urine collection kit in addition to the conventional invitation letter.  Control arm: women receive a conventional invitation letter sent by post to the home address of eligible women recommending them to make an appointment to a doctor or a midwife for the collection of a cervical specimen |
|  | 11b | Criteria for discontinuing or modifying allocated interventions for a given trial participant (eg, drug dose change in response to harms, participant request, or improving/worsening disease) | There are no discontinuity criteria defined since not of relevance for this trial. |
|  | 11c | Strategies to improve adherence to intervention protocols, and any procedures for monitoring adherence (eg, drug tablet return, laboratory tests) | There will be no strategies applied to improve adherence on top of the compared interventions. |
|  | 11d | Relevant concomitant care and interventions that are permitted or prohibited during the trial | Women allocated to an experimental or a control intervention may attend to cervical cancer screening following another procedure than that foreseen in the particular arm. Such events will be disregarded in the per-protocol analyses, where only the number of women will be counted that use the foreseen procedure. However, women who have a screening performed, irrespective whether it is the one foreseen in the trial arm will be included in the intention-to-treat analysis. |
| Outcomes | 12 | Primary, secondary, and other outcomes, including the specific measurement variable (eg, systolic blood pressure), analysis metric (eg, change from baseline, final value, time to event), method of aggregation (eg, median, proportion), and time point for each outcome. Explanation of the clinical relevance of chosen efficacy and harm outcomes is strongly recommended | 1ary outcomes  Participation rates (number of responding / number of invited) in each arm, difference and ratios of participation rates between the control and the two intervention arms.  2ary outcomes   - Contrast in participation between the 2 experimental arms and between women that received a questionnaire or not. - Screen test positivity rates (presence of high-risk HPV and distribution by HPV type and/or of abnormal cytology) in the respective arms and contrasts between arms. - Adherence to follow-up of screen-positive women and contrasts between arms. - Impact of covariates on the participation and adherence to follow-up (age, reimbursement status, geographical area). - Analyse of the obstacles and levers emerging from the speech of the women and health professionals participating in the research on the basis of a thematic analysis and categorical analysis. |
| Participant timeline | 13 | Time schedule of enrolment, interventions (including any run-ins and washouts), assessments, and visits for participants. A schematic diagram is highly recommended (see Figure) | We provided a schematic diagram in the study protocol. |
| Sample size | 14 | Estimated number of participants needed to achieve study objectives and how it was determined, including clinical and statistical assumptions supporting any sample size calculations | The sample size computation was driven by two assessments: difference in proportion (% participation) in the control vs the two experimental arm and the precision of the estimate of follow-up adherence among screen-positive women.  The first sample size computation considered a series of possible assumed participation rates in the control arm (12-14%) and experimental arms (3 to 7% higher than in the control arm) based on cervical cancer screening participation studies in France or in studies included in a recent meta-analyse, completed with plausible ranges of variation. The accepted confidence level was 95% and the power 90%. Entering these parameters, yielded required sample sizes between 588 and 3123 subjects to be enrolled per sample arm.  To estimate the compliance with further follow-up among women with a positive screening test with a 95% confidence interval (CI) width of 10%, assuming compliance rates ranging from 41% to 92% (respectively lowest and highest rates observed in a meta-analysis (Arbyn, 2018), a sample size varying from 1327 to 4,360 women is needed. By pooling the two experimental arms, it will be possible to obtain more precise (more narrow CIs) estimates of the adherence to further follow-up among women with an HPV-positive result on a self-sample.  To conclude enrolment of 3 x 5000 women will allow sufficient power to demonstrate higher efficacy of the experimental compared to the control group and to reach sufficient precision to estimate follow-up compliance. |
| Recruitment | 15 | Strategies for achieving adequate participant enrolment to reach target sample size | No additional strategies to maximise participation in the trial than the control and experimental interventions will be applied, since this would contaminate the measurement of the aimed effect. |
| **Methods: Assignment of interventions (for controlled trials)** | | |  |
| Allocation: |  |  |  |
| Sequence generation | 16a | Method of generating the allocation sequence (eg, computer-generated random numbers), and list of any factors for stratification. To reduce predictability of a random sequence, details of any planned restriction (eg, blocking) should be provided in a separate document that is unavailable to those who enrol participants or assign interventions | The screening organisation will randomise the group of women fulfilling the eligibility criteria in 3 groups using a random number generator. Subsequently a sub-randomisation will be performed within each randomised group.  The randomisation will not be stratified according to covariates. |
| Allocation concealment mechanism | 16b | Mechanism of implementing the allocation sequence (eg, central telephone; sequentially numbered, opaque, sealed envelopes), describing any steps to conceal the sequence until interventions are assigned | The software used for randomisation is the Stata 16.0 random number generator (College Station, TX, USA). |
| Implementation | 16c | Who will generate the allocation sequence, who will enrol participants, and who will assign participants to interventions | The generation of the random allocation will be performed by the IT service appointed by the screening organisation who has experience of randomising groups. |
| Blinding (masking) | 17a | Who will be blinded after assignment to interventions (eg, trial participants, care providers, outcome assessors, data analysts), and how | Given the nature of a participation trial, blinding of the invitations, of the invited women and of the assessment of screen test results on physically different specimens is irrelevant. |
|  | 17b | If blinded, circumstances under which unblinding is permissible, and procedure for revealing a participant’s allocated intervention during the trial | Not of application. |
| **Methods: Data collection, management, and analysis** | | |  |
| Data collection methods | 18a | Plans for assessment and collection of outcome, baseline, and other trial data, including any related processes to promote data quality (eg, duplicate measurements, training of assessors) and a description of study instruments (eg, questionnaires, laboratory tests) along with their reliability and validity, if known. Reference to where data collection forms can be found, if not in the protocol | - The target population will extract thanks to a query in the CRCDC-Pays de la Loire's business software called Zeus d'Osi-Santé. The list of patients will be included first and last names, dates of birth, addresses, and social security numbers to avoid confusion and duplications. - Questionnaires: To construct the questionnaire, we took inspiration from the VALHUDES questionnaire (De Pauw et al, Arch Pub Health 2021). - The interviews and focus groups will be conducted by psychologists who have experience in qualitative research. - The vaginal and urinary self-samples will be analysed at the virology laboratory of the Angers University Hospital using real-time PCR for the search of oncogenic HPV with extended genotyping (BD Onclarity™ HPV test, BD Diagnostics, Burlington, NC). The Onclarity™ assay fulfils requirements for HPV tests in the context of cervical cancer screening. |
|  | 18b | Plans to promote participant retention and complete follow-up, including list of any outcome data to be collected for participants who discontinue or deviate from intervention protocols | Not applicable |
| Data management | 19 | Plans for data entry, coding, security, and storage, including any related processes to promote data quality (eg, double data entry; range checks for data values). Reference to where details of data management procedures can be found, if not in the protocol | The supply and use of these records for the programme have been submitted to the Commission Nationale de l’Informatique et des Libertés, French National Data Protection Authority in September 2021 (ref. 2223607v0).  Screening organisation: The study data will be stored on a directory accessible only by the holder of a clearance. Access to the software used by the CRCDC is secured by authorised access to authorised persons with a personal login and password. The study's correspondence table will be encrypted and deposited by the sponsor's scientific manager on an individual storage space on the server of the CRCDC.  Team of psychologists: The management of interview data will be secured in accordance with the institutional policy for securing digital data implemented by the University of Angers.  Virology laboratory: The management of HPV test data will be secured in accordance with the information data security policy of the University Hospital of Angers. |
| Statistical methods | 20a | Statistical methods for analysing primary and secondary outcomes. Reference to where other details of the statistical analysis plan can be found, if not in the protocol | Standard descriptive analyses and graphical display methods will be applied to characterise the study population and the study results. Stata version 16 (College Station, TX, USA) will be used for statistical analyses. Inference will be made based on 95% confidence intervals and two-sided p-values <0.05 will determine statistical significance.  Participation within and inter-arms will be assessed as a proportion, differences and ratios of proportions and as a cumulative rate. Multivariate analysis with be performed by logistic regression with participation as a dichotomic outcome (yes or no) and by Cox regression with cumulative participation over time as an incidence-like outcome. Multivariate analysis will include inclusion of covariates, with experimental & control arm as main factors in the 1 aray outcomes. |
|  | 20b | Methods for any additional analyses (eg, subgroup and adjusted analyses) | / |
|  | 20c | Definition of analysis population relating to protocol non-adherence (eg, as randomised analysis), and any statistical methods to handle missing data (eg, multiple imputation) | The analysis population will be the women invited to participate in each arm. Number of participants screened / number invited will be the main outcome. A small fraction of non-participants that received the questionnaire may complete the questionnaire. Given their expected small number, no efforts will be made to impute responses towards all non-screened women given. |
| **Methods: Monitoring** | | |  |
| Data monitoring | 21a | Composition of data monitoring committee (DMC); summary of its role and reporting structure; statement of whether it is independent from the sponsor and competing interests; and reference to where further details about its charter can be found, if not in the protocol. Alternatively, an explanation of why a DMC is not needed | Participation in screening, collection of screen tests results and follow-up information among screen test positives will be monitored as usual by the cancer screening organisation. |
|  | 21b | Description of any interim analyses and stopping guidelines, including who will have access to these interim results and make the final decision to terminate the trial | Not of application since the trial will not involve administration of a product. |
| Harms | 22 | Plans for collecting, assessing, reporting, and managing solicited and spontaneously reported adverse events and other unintended effects of trial interventions or trial conduct | Complaints can be captured via the procedures foreseen by the cancer screening organisation. |
| Auditing | 23 | Frequency and procedures for auditing trial conduct, if any, and whether the process will be independent from investigators and the sponsor | The screening organisation will perform the usual quality control regarding the process and performance of cervical cancer screening, which may include occurrence of interval cancers or cancers among screen-positive women with deficient follow-up. |
| Ethics and dissemination | | |  |
| Research ethics approval | 24 | Plans for seeking research ethics committee/institutional review board (REC/IRB) approval | The CapU4 study protocol received approval from the French “Sud-Est I Ethics Committee” (2021-123, 25/11/2021, France). |
| Protocol amendments | 25 | Plans for communicating important protocol modifications (eg, changes to eligibility criteria, outcomes, analyses) to relevant parties (eg, investigators, REC/IRBs, trial participants, trial registries, journals, regulators) | Significant changes to the protocol will be submitted to the French Ethics Committee "Sud-Est I" before modification. Investigators and trial participants will be informed of these modifications if necessary. |
| Consent or assent | 26a | Who will obtain informed consent or assent from potential trial participants or authorised surrogates, and how | Women's return of the vaginal and urinary self-samples will constitute consent for HPV testing.  Team of psychologists: Written informed consent to the audio recording of the interviews and anonymised digital transcripts will be obtained from the women who will participate in the individual semi-structured interviews and/or focus groups. |
|  | 26b | Additional consent provisions for collection and use of participant data and biological specimens in ancillary studies, if applicable | Not applicable |
| Confidentiality | 27 | How personal information about potential and enrolled participants will be collected, shared, and maintained in order to protect confidentiality before, during, and after the trial | The supply and use of these records for the programme have been submitted to the Commission Nationale de l’Informatique et des Libertés, French National Data Protection Authority in September 2021 (ref. 2223607v0).  The data are collected and centralized by the CRCDC Pays de la Loire (screening organisation). A correspondence table is established separately and is kept for a period of 2 years after publication. After that, only pseudonomised data will be kept. Data entry will be performed by the investigator or a person mandated by him/her in the study database.  The team of psychologists will be responsible for the management of the data related to the semi-structured interviews/focus groups. The interviews and focus groups are digitally recorded after information about the purposes of the research and the completion of a consent form. Only they will have access to the content of the interviews. They will provide the CRCDC (screening organisation) with an analysis that summarizes what was said and does not allow for the re-identification of the person concerned.  The screening organisation will provide the unit of of Cancer Epidemiology (Sciensano, Brussels, Belgium) (statisticians) with the pseudonymized database of the study for statistical analysis. |
| Declaration of interests | 28 | Financial and other competing interests for principal investigators for the overall trial and each study site | Sciensano, the employer of MA and HD,P has received support from the Horizon 2020 Framework Programme for Research and Innovation of the European Commission, through the RISCC Network (Grant No. 847845); Haute Autorité de la Santé (Paris, France); New South Wales Cancer Council (Sydney, Australia) and VALGENT & VALHUDES, which are researcher-induced frameworks for comparison and validation of HPV tests applied on clinician-taken and self-samples (see Arbyn et al, J Clin Virol 2016 and 2018).  The other investigators declare no financial or competing interests. |
| Access to data | 29 | Statement of who will have access to the final trial dataset, and disclosure of contractual agreements that limit such access for investigators | The study sponsor will have access to the final trial data set. |
| Ancillary and post-trial care | 30 | Provisions, if any, for ancillary and post-trial care, and for compensation to those who suffer harm from trial participation | / |
| Dissemination policy | 31a | Plans for investigators and sponsor to communicate trial results to participants, healthcare professionals, the public, and other relevant groups (eg, via publication, reporting in results databases, or other data sharing arrangements), including any publication restrictions | The results of the trial will be reported in the form of a research publication. |
|  | 31b | Authorship eligibility guidelines and any intended use of professional writers | All investigators or persons actively participating in the CapU4 study will be eligible as authors. |
|  | 31c | Plans, if any, for granting public access to the full protocol, participant-level dataset, and statistical code | / |
