# Supplementary material for: Study Protocol: Randomised Controlled Trial Assessing the Efficacy of Strategies Involving Self-Sampling in Cervical Cancer Screening
Source: Int J Public Health. 2022 Feb 24;67:1604284. doi: 10.3389/ijph.2022.1604284 (PMC8907121; doi:10.3389/ijph.2022.1604284)
Supplement: Supplementary file 2 [file DataSheet1.DOCX]

**Supplementary Material**

**Urine self-sampling questionnaire**

**Before self-sampling**

*Dear Madam,*

*We propose you to participate in a study to improve cervical cancer screening. Please take 2 minutes to answer the following questions.*

*The term cervical smear refers to a gynecological specimen taken by a health care professional.*

Date of completion of the questionnaire: .../.../…

| U1. How old are you? | … years |
| --- | --- |
| U2.a Have you ever had a gynecological specimen?  U2.b If yes, can you estimate the date of the last gynecological specimen?  U2.c If you have never had a gynecological specimen, indicate the reason: | □ yes □ no  □ < 1 year □ 1-3 years □ 4-5 years □ 5 years or more  □ I don't know for how many years  □ I no longer have a uterus (total hysterectomy)  □ It was never proposed to me  □ I refused this sample  □ I did not know  □ I have never had sexual relations  □ Other reason: … |

U3. You did not have a gynecological specimen because ...

| U3.a You don't have the time to go to your general practitioner/gynecologist/midwife | □ yes □ no □ no opinion |
| --- | --- |
| U3.b The appointment delay is too long to go to your general practitioner/gynecologist/midwife | □ yes □ no □ no opinion |
| U3.c The sampling is painful | □ yes □ no □ no opinion |
| U3.d The sampling makes you uncomfortable | □ yes □ no □ no opinion |
| U3.e I am anxious to receive a positive result | □ yes □ no □ no opinion |

U4. Before agreeing to participate in this study ...

| U4.a … Have you ever heard of cervical cancer? | □ yes □ no □ no opinion |
| --- | --- |
| U4.b … Did you know that cervical cancer is caused by the human papillomavirus (= HPV), a sexually transmitted virus? | □ yes □ no □ no opinion |

| U5.a Do you think that self-sampling will be better accepted by women who do not regularly go to a general practitioner/gynecologist/midwife for a gynecological specimen? | □ yes □ no □ no opinion |
| --- | --- |
| U5b. I have more confidence in having a gynecological specimen taken by a doctor/midwife than a home self-sampling | □ yes □ no □ no opinion |
| U5c. I had a bad experience with a gynecological specimen, I would prefer a home self-sampling | □ yes □ no □ no opinion |

**Questionnaire after urine self-sampling**

U6. What is your opinion about the urine sample you took at home?

|  | Totally agree | Partly agree | Don’t agree | No opinion |
| --- | --- | --- | --- | --- |
| U6a. The instructions for self-sampling was clear |  |  |  |  |
| U6b. The sampling was easy |  |  |  |  |
| U6c. I found the sampling unpleasant |  |  |  |  |
| U6d. The sampling was painful |  |  |  |  |
| U6e. I think that I executed the sampling correctly |  |  |  |  |
| U6f. I would recommend this to my friends/family |  |  |  |  |
| U6g. I find urine sampling at home easier than a Pap smear, because then I don’t need to go to the doctor/midwife |  |  |  |  |
| U6h. I think it would be good to have an appointment with my doctor/midwife after this self-sampling to have explanations on the interpretation of the results |  |  |  |  |

U7. What would you prefer at your next cervical cancer screening?

|  | Totally agree | Partly agree | Don’t agree | No opinion |
| --- | --- | --- | --- | --- |
| U7a. Collect a urine sample myself with the self-sampling kit |  |  |  |  |
| U7b. Have a gynecological sample taken by my gynecologist |  |  |  |  |
| U7c. Have a gynecological sample taken by my general practitioner |  |  |  |  |
| U7d. Have a gynecological sample taken by my midwife |  |  |  |  |

U8. What is your preference for a future self-sampling screening?

□ Receive this kit at home

□ Obtain this kit from my general practitioner/gynecologist/midwife.

□ Obtain this kit from a pharmacy

□ No opinion

U9. In order to allow other women to have access to self-sampling in the best possible conditions, would you accept a totally anonymous telephone interview with a professional?

□ no

□ yes and I can be reached at this phone number: .. - .. - .. - .. - ..

**Vaginal self-sampling questionnaire**

**Before self-sampling**

*Dear Madam,*

*We propose you to participate in a study to improve cervical cancer screening. Please take 2 minutes to answer the following questions.*

*The term cervical smear refers to a gynecological specimen taken by a health care professional.*

Date of completion of the questionnaire: .../.../…

| V1. How old are you? | … years |
| --- | --- |
| V2.a Have you ever had a gynecological specimen?  V2.b If yes, can you estimate the date of the last gynecological specimen?  V2.c If you have never had a gynecological specimen, indicate the reason: | □ yes □ no  □ < 1 year □ 1-3 years □ 4-5 years □ 5 years or more  □ I don't know for how many years  □ I no longer have a uterus (total hysterectomy)  □ It was never proposed to me  □ I refused this sample  □ I did not know  □ I have never had sexual relations  □ Other reason: … |

V3. You did not have a gynecological specimen because ...

| V3.a You don't have the time to go to your general practitioner/gynecologist/midwife | □ yes □ no □ no opinion |
| --- | --- |
| V3.b The appointment delay is too long to go to your general practitioner/gynecologist/midwife | □ yes □ no □ no opinion |
| V3.c The sampling is painful | □ yes □ no □ no opinion |
| V3.d The sampling makes you uncomfortable | □ yes □ no □ no opinion |
| V3.e I am anxious to receive a positive result | □ yes □ no □ no opinion |

V4. Before agreeing to participate in this study ...

| V4.a … Have you ever heard of cervical cancer? | □ yes □ no □ no opinion |
| --- | --- |
| V4.b … Did you know that cervical cancer is caused by the human papillomavirus (= HPV), a sexually transmitted virus? | □ yes □ no □ no opinion |

| V5.a Do you think that self-sampling will be better accepted by women who do not regularly go to a general practitioner/gynecologist/midwife for a gynecological specimen? | □ yes □ no □ no opinion |
| --- | --- |
| V5b. I have more confidence in having a gynecological specimen taken by a doctor/midwife than a home self-sampling | □ yes □ no □ no opinion |
| V5c. I had a bad experience with a gynecological specimen, I would prefer a home self-sampling | □ yes □ no □ no opinion |

**Questionnaire after vaginal self-sampling**

V6. What is your opinion about the vaginal self-sampling with the cotton swab that you performed at home?

|  | Totally agree | Partly agree | Don’t agree | No opinion |
| --- | --- | --- | --- | --- |
| V6a. The instructions for self-sampling was clear |  |  |  |  |
| V6b. The sampling was easy |  |  |  |  |
| V6c. I found the sampling unpleasant |  |  |  |  |
| V6d. The sampling was painful |  |  |  |  |
| V6e. I think that I executed the sampling correctly |  |  |  |  |
| V6f. I would recommend this to my friends/family |  |  |  |  |
| V6g. I find vaginal sampling at home easier than a Pap smear, because then I don’t need to go to the doctor/midwife |  |  |  |  |
| V6h. I think it would be good to have an appointment with my doctor/midwife after this self-sampling to have explanations on the interpretation of the results |  |  |  |  |

V7. What would you prefer at your next cervical cancer screening?

|  | Totally agree | Partly agree | Don’t agree | No opinion |
| --- | --- | --- | --- | --- |
| V7a. Collect a vaginal sample myself with the self-sampling kit |  |  |  |  |
| V7b. Have a gynecological specimen taken by my doctor/midwife with the vaginal self-sampling kit |  |  |  |  |
| V7c. Have a gynecological sample taken by my gynecologist |  |  |  |  |
| V7d. Have a gynecological sample taken by my general practitioner |  |  |  |  |
| V7e. Have a gynecological sample taken by my midwife |  |  |  |  |

V8. What is your preference for a future self-sampling screening?

□ Receive this kit at home

□ Obtain this kit from my general practitioner/gynecologist/midwife.

□ Obtain this kit from a pharmacy

□ No opinion

V9. In order to allow other women to have access to self-sampling in the best possible conditions, would you accept a totally anonymous telephone interview with a professional?

□ no

□ yes and I can be reached at this phone number: .. - .. - .. - .. - ..

**Control arm questionnaire**

**Before gynecological specimen by a health professional**

*Dear Madam,*

*We suggest that you make an appointment with your doctor/midwife to have a gynecological specimen for cervical cancer screening. In order to improve this screening, even if you do not plan to make an appointment for this sample, please take 1 minute to answer the following questions.*

*The term cervical smear refers to a gynecological specimen taken by a health care professional.*

Date of completion of the questionnaire: .../.../…

| C1. How old are you? | … years |
| --- | --- |
| C2.a Have you ever had a gynecological specimen?  C2.b If yes, can you estimate the date of the last gynecological specimen?  C2.c If you have never had a gynecological specimen, indicate the reason: | □ yes □ no  □ < 1 year □ 1-3 years □ 4-5 years □ 5 years or more  □ I don't know for how many years  □ I no longer have a uterus (total hysterectomy)  □ It was never proposed to me  □ I refused this sample  □ I did not know  □ I have never had sexual relations  □ Other reason: … |

C3. You did not have a gynecological specimen because ...

| C3.a You don't have the time to go to your general practitioner/gynecologist/midwife | □ yes □ no □ no opinion |
| --- | --- |
| C3.b The appointment delay is too long to go to your general practitioner/gynecologist/midwife | □ yes □ no □ no opinion |
| C3.c The sampling is painful | □ yes □ no □ no opinion |
| C3.d The sampling makes you uncomfortable | □ yes □ no □ no opinion |
| C3.e I am anxious to receive a positive result | □ yes □ no □ no opinion |

C4a. Following this letter of invitation to have a gynecological specimen, would you like to make an appointment with your doctor/midwife to have the gynecological specimen taken? □ yes □ no

C4b. If not, indicate the reason:

□ You don't have the time to go to your general practitioner/gynecologist/midwife

□ The appointment delay is too long to go to your general practitioner/gynecologist/midwife

□ The sampling is painful

□ The sampling makes you uncomfortable

□ I am anxious to receive a positive result

□ Other reason(s) (specify in box)

C5. Before receiving this letter of invitation to have a gynecological specimen ...

| C5.a … Have you ever heard of cervical cancer? | □ yes □ no □ no opinion |
| --- | --- |
| C5.b … Did you know that cervical cancer is caused by the human papillomavirus (= HPV), a sexually transmitted virus? | □ yes □ no □ no opinion |
